# Anatomical Versus Functional Methods for Hippocampal Subfields Segmentation: Letter to the Editor Regarding the Manuscript “Altered Hippocampal Subfields Functional Connectivity in Benign Paroxysmal Positional Vertigo Patients With Residual Dizziness: A Resting‐State fMRI Study”

**DOI:** 10.1111/cns.70521

**Published:** 2025-07-23

**Authors:** Kwangsun Yoo, Eek‐Sung Lee

**Affiliations:** ^1^ Department of Digital Health, Samsung Advanced Institute for Health Sciences and Technology Sungkyunkwan University Seoul Republic of Korea; ^2^ AI Research Center, Research Institute for Future Medicine Samsung Medical Center Seoul Republic of Korea; ^3^ Department of Neurology Soonchunhyang University Bucheon Hospital Bucheon Republic of Korea

**Keywords:** functioanl connectivity, functional MRI, hippocampus, vestibular disorder

Dear Editor,

1

We have read with great interest the recent article by Chen et al. titled “Altered Hippocampal Subfields Functional Connectivity in Benign Paroxysmal Positional Vertigo Patients With Residual Dizziness: A Resting‐State fMRI Study” published in CNS Neuroscience & Therapeutics [1]. This study provides valuable insights into the role of hippocampal connectivity in the mechanism of residual dizziness following BPPV. We would like to offer methodological considerations regarding the authors' approach to hippocampal subfield segmentation.

There are two primary methods for hippocampal subfield segmentation: an anatomical method based on cytoarchitectonic probabilistic atlases, and a functional method based on the anterior–posterior (A‐P) axis of the hippocampus. While the authors used anatomical segmentation with cytoarchitectonic atlases, this approach is limited by current imaging resolution [2]. Their analysis involved hippocampal subfield segmentation on 3 T MRI images, followed by functional connectivity analysis using specific subfield ROIs as seeds. Since most hippocampal layers except the dentate gyrus are less than 2 mm thick, it is difficult to avoid the partial volume effect [3]. Additionally, the authors defined Cornu Ammonis (CA) as a single region, despite distinct cellular properties and connectivity patterns across CA1 to CA3 subfields [4, 5]. This unified characterization of CA subregions introduces methodological concerns, particularly in the form of partial volume effects and signal mixing between subfields, which could potentially generate spurious connectivity findings [6].

From a functional perspective, the hippocampus exhibits a clear anterior–posterior axis organization, as demonstrated by recent research [7] that challenges the traditional view of this structure as uniformly organized. Single‐nucleus RNA sequencing studies have revealed systematic gradients in gene expression, demonstrating distinct cellular and molecular signatures that differentiate the anterior from the posterior hippocampus. This molecular heterogeneity is paralleled by differential connectivity patterns, with the anterior hippocampus linking to limbic and prefrontal networks for emotional and memory processing, while the posterior portion connects with parietal and occipital cortices for spatial and visual functions [8]. In support of this perspective, our research in persistent postural‐perceptual dizziness (PPPD) patients validates the A‐P axis‐based segmentation approach, revealing decreased functional connectivity between the anterior hippocampus and several regions [9]. Task fMRI studies have further corroborated these findings, demonstrating distinct patterns of activation and interregional synchrony along the hippocampal long axis during cognitive tasks [10].

In conclusion, while defining ROIs based on cytoarchitectonic atlases is a widely used method in hippocampal neuroimaging studies, its limitations in spatial resolution of fMRI may impact functional connectivity analyses. The A‐P axis‐based approach, reflecting the hippocampus's functional and molecular heterogeneity, could better characterize alterations of brain networks in vestibular disorders.

## Conflicts of Interest

The authors declare no conflicts of interest.

## Data Availability

Data sharing not applicable to this article as no datasets were generated or analysed during the current study.
